# The Limited Impact of Exposure Duration on Holistic Word Processing

**DOI:** 10.3389/fpsyg.2016.00646

**Published:** 2016-06-06

**Authors:** Changming Chen, Najam ul Hasan Abbasi, Shuang Song, Jie Chen, Hong Li

**Affiliations:** ^1^Key Laboratory of Cognition and Personality, Ministry of Education, Faculty of Psychology, Southwest UniversityChongqing, China; ^2^Research Center for Brain Function and Psychological Science, Shenzhen UniversityShenzhen, China; ^3^Department of Psychology, International Islamic UniversityIslamabad, Pakistan; ^4^Department of Psychology, University of SindhJamshoro, Pakistan; ^5^Research Center of Brain and Cognitive Neuroscience, Liaoning Normal UniversityDalian, China; ^6^Department of Psychology, Hunan Normal UniversityChangsha, China

**Keywords:** composite task, holistic processing, word recognition, Chinese character, signal detection theory

## Abstract

The current study explored the impact of stimuli exposure duration on holistic word processing measured by the complete composite paradigm (CPc paradigm). The participants were asked to match the cued target parts of two characters which were presented for either a long (600 ms) or a short duration (170 ms). They were also tested by two popular versions of the CPc paradigm: the “early-fixed” task where the attention cue was visible from the beginning of each trial at a fixed position, and the “delayed-random” task where the cue showed up after the study character at random locations. The holistic word effect, as indexed by the alignment × congruency interaction, was identified in both tasks and was unaffected by the stimuli duration in both tasks. Meanwhile, the “delayed-random” task did not bring about larger holistic word effect than the “early-fixed” task. These results suggest the exposure duration (from around 150 to 600 ms) has a limited impact on the holistic word effect, and have methodological implications for experiment designs in this field.

## Introduction

Research on word recognition has a century-long history (Cattell, 1886; Pillsbury, 1897), but consensus has not been reached on the basic question “if visual words are processed holistically” (Wong et al., 2012). Although accumulating evidence like the word superiority effect (Cattell, 1886; Reicher, 1969) and the cAsE altErNaTiOn effect (Allen et al., 1995) suggests that words are processed as a whole unit, there are still voices arguing for “part-based” recognition of visual words (Bouwhuis and Bouma, 1979; Farah, 1990; Pelli et al., 2003; Martelli et al., 2005; Wilson and Taylor, 2009).

**Figure 1:**
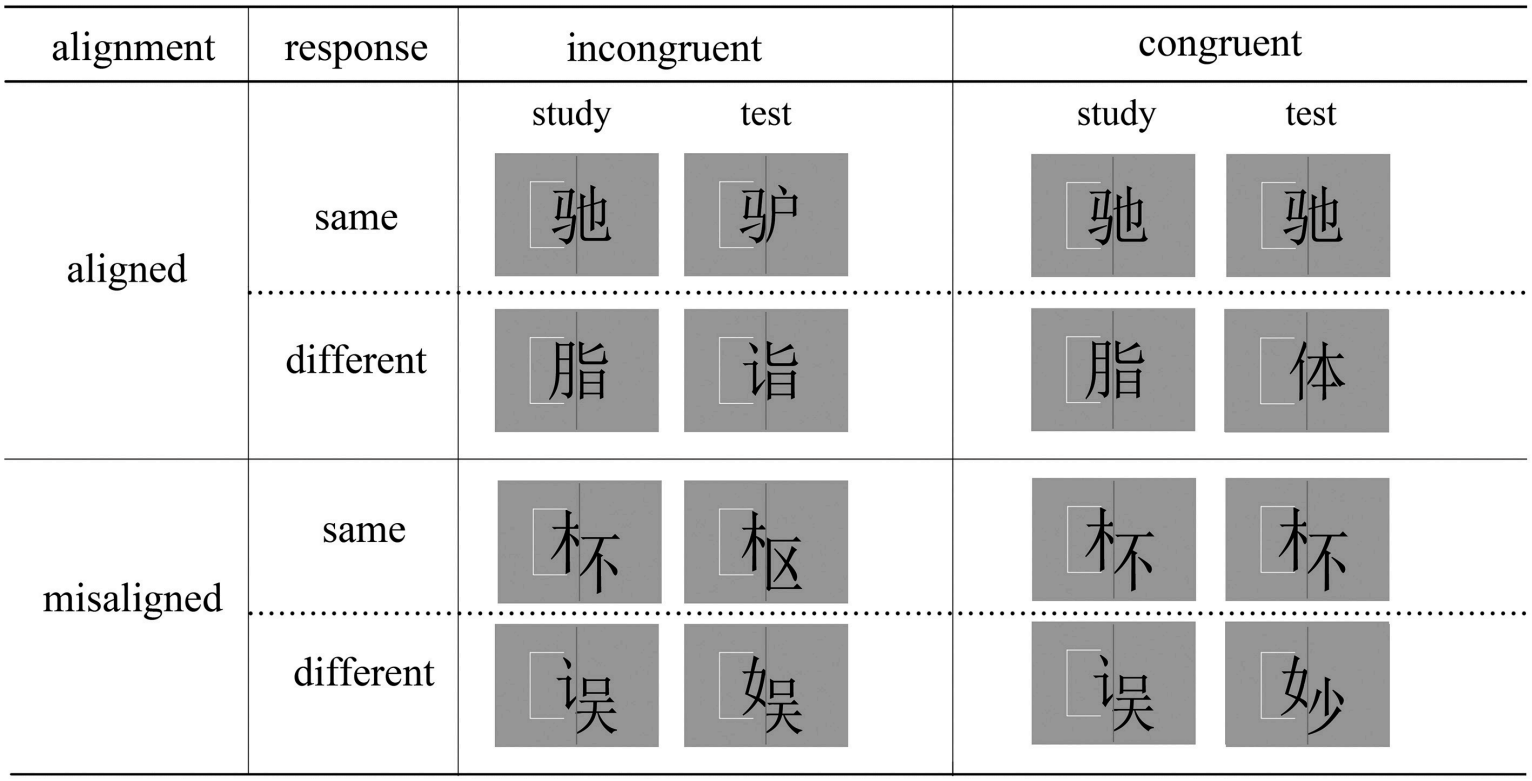
**Trial types and sample Chinese characters in the complete composite task**. The alignment (aligned, misaligned) × congruency (congruent, incongruent) × response (same, different) combination results into 8 trial types. Participants were asked to match the target parts (cued by the white bracket) of two sequential characters in each trial.

In the last decade, some researchers investigated holistic word processing using a task called the “complete composite paradigm” (“CPc paradigm” hereafter, Gauthier and Bukach, 2007; Richler and Gauthier, 2013). In this task, holistic processing is defined as the obligatory attention to all parts of a stimulus despite instructions to focus only on one part (Wong et al., 2011). Participants are asked to match the target parts (e.g., the left halves) of two stimuli (e.g., words) while ignoring the irrelevant parts (Figure 1). *In the congruent trials*, both the target and irrelevant parts are identical, or both are different. *In the incongruent trials*, the two parts in one location are identical but those in the other location are different. In many studies a misalignment manipulation is also included: the target and irrelevant parts are either aligned or misaligned, and holistic processing could be indexed by an alignment × congruency interaction with smaller congruency effect in the misaligned trials (Cheung et al., 2008; Wong et al., 2009b; Richler and Gauthier, 2014). With its advantage in controlling response bias (Gauthier and Bukach, 2007; Richler and Gauthier, 2014), the CPc paradigm has been used to explore the involvement of holistic processing in a wide-range of categories (Gauthier et al., 1998, 2000; Gauthier and Curby, 2005; Wong et al., 2009a; Wong and Gauthier, 2010; Boggan et al., 2012).

Using this paradigm, the researchers observed holistic processing in both English words (Wong et al., 2011) and Chinese characters (Leung, 2012; Wong et al., 2012). In the Chinese writing system, holistic effect could be observed when the study and test characters were presented sequentially, and when they were presented simultaneously. It could also be observed in both the left-right configured and top-bottom configured characters, irrespective of the character structure (Wong et al., 2012). These results have led to the proposal that holistic processing is a marker of expertise for both faces and non-face categories, including visual words (Wong et al., 2012).

The holistic word effect has been well-established by the CPc paradigm. As far as we know, most of previous studies focused on the role of reading experience (Hsiao and Cottrell, 2009; Wong et al., 2011, 2012; Leung, 2012; Zhong, 2012; Tso et al., 2014), and little is known about how it would be modulated by other factors. In the current study, we investigated how the holistic word effect measured by the CPc paradigm would be affected by stimuli exposure duration. This could help reveal the dynamics of holistic word processing. In a previous study on face recognition, Richler et al. parametrically varied the stimulus duration from 17 ms in the shortest presentation condition to 800 ms in the longest presentation condition. The holistic effect, as indexed by the congruency effect, was observed when faces were presented very briefly (for 50 ms). From 50 ms onwards it was neither affected by the duration of the study face, nor by the duration of test face (Richler et al., 2009b). That study made a systematic exploration of the relationship between the holistic effect and exposure duration. However, several issues are worth noting. First, Richler's study investigated the holistic face effect. Words and faces differ in multiple aspects, from their physical properties to their underlying cognitive and neural mechanisms (McKone et al., 2007; Kanwisher, 2010). Second, Richler et al. used a CPc task without the misalignment manipulation, but in studies on non-face categories misaligned trials are recommended (Richler and Gauthier, 2014) because the congruency effect with no alignment by congruency interaction could be brought about by strategies other than holistic processing (Wong and Gauthier, 2010; Richler and Gauthier, 2014). As a result, it is unknown if the above mentioned findings could be generalized to visual words.

From the methodological perspective, in previous studies when the duration of word composites was 400 ms (Wong et al., 2011) or longer (Hsiao and Cottrell, 2009; Zhong, 2012), the participants typically achieved very high performance levels. This might compromise the studies' validity by creating a ceiling effect. In later studies, certain methods were used to ensure the validity and power of experimental design. For example, Wong and colleagues introduced a response deadline (e.g., 700 ms for expert readers) such that participants responded with considerable time pressure and ceiling/floor effects could be eliminated (Wong et al., 2012). As an alternative, Hsiao and Cottrell (2009) chose to shorten the stimuli presentation (to 500 ms) and reduce the stimuli contrast level, they decreased expert readers' average discrimination sensitivity from 0.978 to 0.948 (p. 458). However, performance under the latter context still seemed high. So in the current study we also wanted to explore if the ceiling effect risk could be avoided by a much shorter duration, and how the holistic word effect would be affected as duration decreases.

To this end, we asked two groups of native Chinese college students to finish the complete composite task. In one group, the characters were presented for a long duration (600 ms) as in previous studies, and for the other group the characters were presented more briefly (170 ms). Moreover, we examined participants with two of the most-used versions of the CPc paradigm. In the CPc paradigm, a cue (e.g., a white bracket) is usually used to notify participants about which parts they are to compare, and two versions of CPc paradigms could be found according to how the cue has been manipulated. In one version, to ensure that the participants have the best knowledge of attention locus, the cue is presented early at the beginning of each trial, and is visible in both the study and test stimuli. It also remains at the same location across a block of trials (“the early-fixed method”). In the other one, the cue shows up only after the study stimulus has disappeared, and its location is randomized across trials (“the delayed-random method,” Wong et al., 2011, 2012). Testing the participants under these two CPc tasks could provide more information about the effect of duration on the holistic word effect, and be methodologically instrumental in helping researchers to decide the experimental design.

## “Early-fixed cueing” task

In this task, we investigated the effect of exposure duration when participants were cued by the “early-fixed” method.

### Participants and ethics

Eighty-two native Chinese undergraduate or graduate students (31 males, mean age = 21.26 ± 2.02 years old) were paid for participation. All of them had normal or corrected-to-normal visual acuity, none of them had any history of brain injury. They were divided randomly into two groups, with 41 people each. The two groups were equal in gender distribution, χ^2^ = 0.47, *p* > 0.50, and age, *t*_(80)_ = −1.60, *p* > 0.10. The study and test characters were presented for 600 ms in one group, and 170 ms in the other. The study was approved by the Ethics Committee of Southwest University in China. Informed consents were obtained in written form from all the participants.

### Materials

Twenty-eight real Chinese characters of left-right configuration were used in this task **(Appendix 1)**. Given that radicals play an important functional role in Chinese word recognition (Feldman and Siok, 1997; Perfetti et al., 2005), the 28 characters were carefully chosen so that they were made from 14 common left and 14 common right radicals with each radical used equally twice. This minimized the variance of the frequency with which the radicals showed up across experimental conditions, balanced the familiarity effect and minimized confounds from non-visual properties. The characters were all in SONG font against a gray background with a visual angle of 3.72° × 3.72°. In the aligned stimuli, the left and right halves in each character were separated by a center-interposed vertical line (3 pixels). The misaligned stimuli were made from the aligned characters by nudging the left/right radical downward by 50 pixels (See Figure 1 for sample characters and experimental conditions).

### Experimental procedure

**Figure 2:**
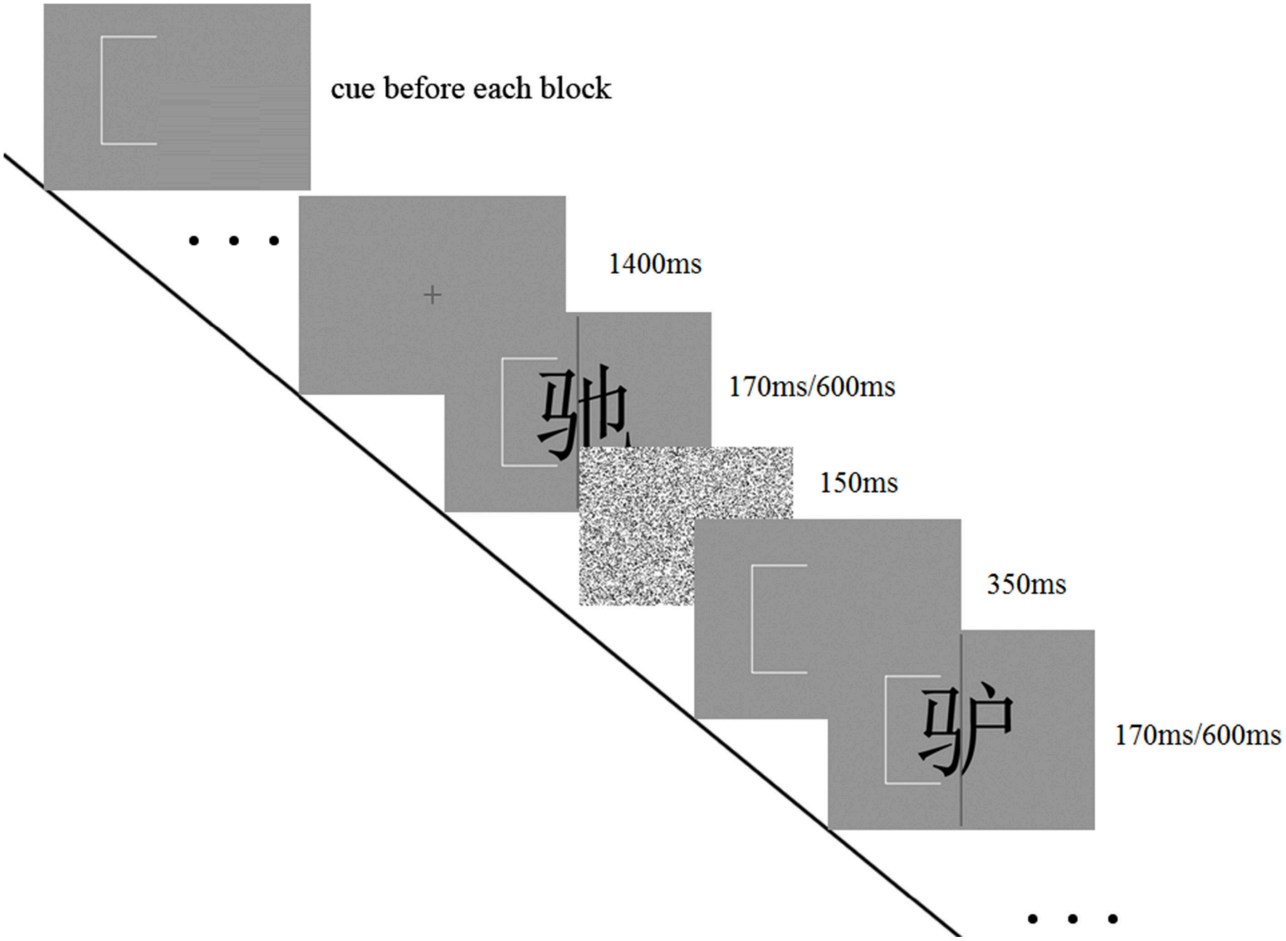
**Trial sequence in the “early-fixed cueing” task**.

The task consisted of four blocks with a total of 224 trials. There were 28 trials for each of the eight conditions (aligned/misaligned × congruent/incongruent × same/different response). The order of conditions was randomized in each block. At the beginning of each block, a white bracket showed up either in the left or right half of the screen to notify participants about which half they should compare. The cue was constantly at the same position for trials within each block, but followed a “left-right-right-left” order among blocks. Each trial began with a fixation cross for a random duration (1400 ms on average), then the study character (with the cue), a mask (150 ms), the cue bracket again (350 ms) and finally the test character (with the cue). All the characters were presented at the center of the screen. To make the task challenging (Wong et al., 2012), the participants were required to make responses within 1500 ms after the onset of the test character. The 1500 ms response limit was decided based on our pilot study. They could have a break after a block, and proceed to the next block whenever they were ready (see Figure 2).

The participants were seated in a quiet room about 80 cm from the monitor. A practice session with at least 16 trials was used before the formal test. Participants could not proceed to the formal test until they achieved at least 85% accuracy in practice. In the complete composite paradigm, theoretically the participants can get an overall accuracy of 75% even when they do not follow the “part-part matching” instruction but use a different strategy like “whole-whole matching”. In light of this, we chose a threshold higher than 75% to ensure that participants understood the task. Characters used during practice were not used in the formal test.

### Data analysis

Participants were discarded if they demonstrated an obvious response bias or extreme response speed. The detailed criteria for participant exclusion were as follow: (1) The overall accuracy for one type of response (e.g., the “same” trials) was higher than 0.75 but that for another response (e.g., the “different”) was lower than 0.25, (2) the average reaction time was shorter than 300 ms in any of the 2 (Cue: early, delayed) × 2 (alignment: aligned, misaligned) × 2 (Congruency: congruent, incongruent) combinations. For the remaining participants, trials that met any of the following criteria were discarded: (1) no response, (2) reaction time less than 200 ms or more than 1500 ms or beyond three standardized variations away from the individual mean. These criteria were decided based on results in previous publications (Hsiao and Cottrell, 2009; Wong et al., 2011) as well as our preliminary tests. None of the participants were discarded in the two tasks. For the remaining participants, 2.69% of the trials were discarded in the 600-ms task and 2.81% were discarded in the 170-ms task.

Discrimination index and reaction time were estimated individually for each of the alignment by congruency combinations, and then submitted to group analysis. The discrimination index was calculated by the following formula (Stanislaw and Todorov, 1999): $A\prime =0.5+sign(H-F)\frac{{\left(H\;-\;F\right)}^{2}+|H\;-\;F|}{4\max(H,\;F)\;-\;4HF}$, where the *H* and *F* indicated hit rate and false alarm rate, respectively. The index *A*' has been suggested to be more robust than d' to the influence of response bias (Verde et al., 2006; Richler et al., 2008).

A 2 (duration: long, short) × 2 (alignment: aligned, misaligned) × 2 (congruency: congruent, incongruent) repeated measurements ANOVA was first conducted to explore the effect of duration on holistic effect. Given that the alignment × congruency interaction implies holistic processing in non-face categories, we were especially interested in if the three-way interaction was significant. We also reported the results of two-way repeated measurements ANOVAs to reveal the detailed response profile under each duration, in hope to enrich the information of this article. These statistics can provide data sources for future meta-analysis in this field (Richler and Gauthier, 2014).

### Results

**Figure 3:**
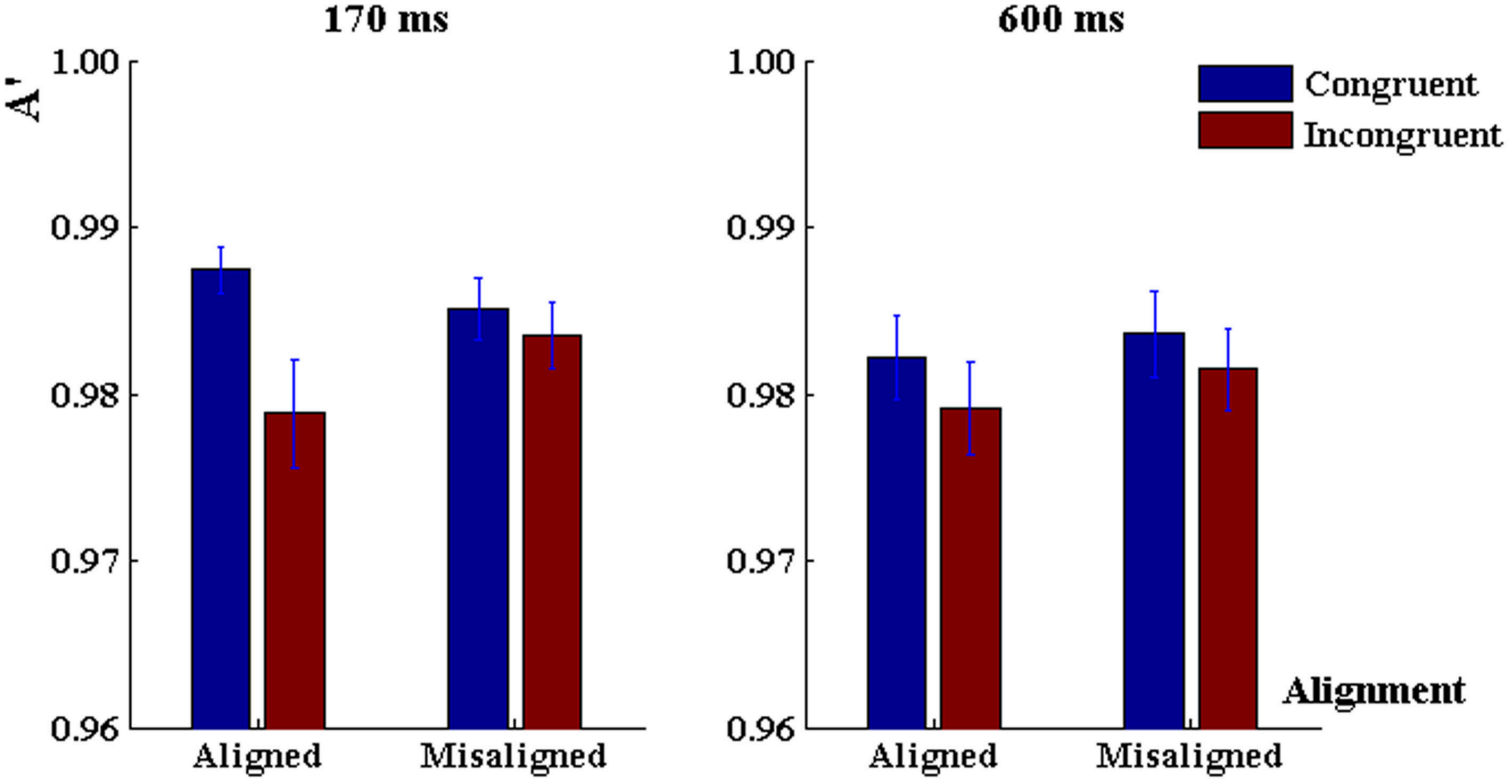
**Discrimination sensitivity in the “early-fixed cueing” task when the duration of character presentation was 170 ms (left panel) and 600 ms (right panel)**. Error bars represent 1 SEM.

The 3-way ANOVA on discrimination sensitivity (Figure 3) revealed a significant main effect of congruency, *F*_(1, 80)_ = 8.83, *p* < 0.001, $\eta_{p}^{2}=$ 0.1, a marginally significant interaction between alignment and congruency, *F*_(1, 80)_ = 3.06, *p* = 0.08, $\eta_{p}^{2}\;=$ 0.04, but no main effect of alignment, *F*_(1, 80)_ = 0.98, *p* > 0.30. There was no main effect of exposure duration, *F*_(1, 80)_ = 1.13, *p* > 0.30, no duration by alignment interaction, *F*_(1, 80)_ = 0.13, *p* = 0.72, no duration by congruency either, *F*_(1, 80)_ = 0.91, *p* > 0.30. Importantly, we did not find a three-way interaction among these factors either, *F*_(1, 80)_ = 1.58, *p* > 0.20.

**Figure 4:**
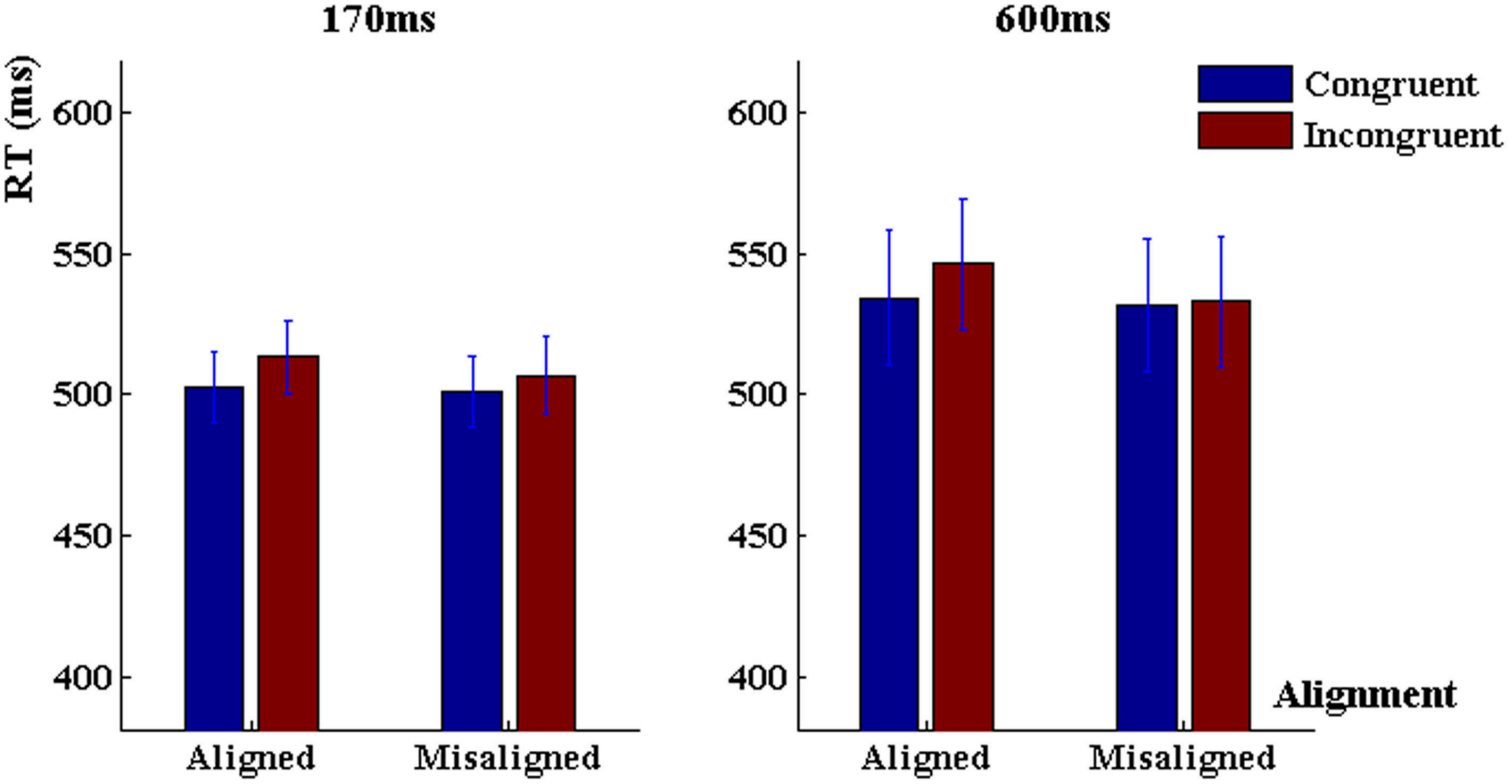
**Reaction time in the “early-fixed cueing” task when character presentation was 170 ms (left panel) and 600 ms (right panel)**. Error bars represent 1 SEM.

The three-way ANOVA on reaction time revealed a significant main effect of congruency, *F*_(1, 80)_ = 31.63, *p* < 0.001, $\eta_{p}^{2}=$ 0.28, main effect of alignment, *F*_(1, 80)_ = 13.87, *p* < 0.001, $\eta_{p}^{2}\;=$ 0.15. Generally, reaction time was longer in the incongruent trials and the aligned trials. Meanwhile, there was a significant alignment × congruency interaction, *F*_(1, 80)_ = 8.96, *p* < 0.01, $\eta_{p}^{2}\;=$ 0.10, and a marginally significant interaction between exposure duration and alignment, *F*_(1, 80)_ = 3.22, *p* < 0.08, $\eta_{p}^{2}$ = 0.04. The reaction time was significantly shorter in the congruent trials than the incongruent trials when characters were aligned (*p* < 0.001, Tukey HSD corrected, “Tukey HSD” hereafter), but did not differ when they were misaligned (*p* > 0.10, Tukey HSD). However, there was no three-way interaction among duration, alignment and congruency, *F*_(1, 80)_ = 0.39, *p* > 0.50, $\eta_{p}^{2}$ < 0.005, nor a main effect of duration, *F*_(1, 80)_ = 0.84, *p* > 0.30, nor a duration × congruency interaction, *F*_(1, 80)_ = 0.22, *p* > 0.60 (Figure 4).

#### 600 ms

First, we conducted 2 (alignment) × 2 (congruency) repeated-measurement ANOVAs to characterize the response profile under the 600-ms word presentation. Analysis of discrimination sensitivity did not reveal significant main effect of alignment, *F*_(1, 40)_ = 0.68, *p* = 0.41, nor a main effect of congruency, *F*_(1, 40)_ = 2.21, *p* = 0.14, nor an alignment × congruency interaction, *F*_(1, 40)_ = 0.10, *p* = 0.75, $\eta_{p}^{2}$ = 0.005 (Figure 3). On reaction time there was a significant main effect of alignment, *F*_(1, 40)_ = 12.96, *p* < 0.001, $\eta_{p}^{2}$ = 0.24, a main effect of congruency, *F*_(1, 40)_ = 13.32, *p* < 0.001, $\eta_{p}^{2}$ = 0.25, and an alignment × congruency interaction, *F*_(1, 40)_ = 6.80, *p* = 0.01, $\eta_{p}^{2}\;=$ 0.15 (Figure 4).

#### 170 ms

Analysis of discrimination sensitivity in the 170-ms task revealed no main effect of alignment, *F*_(1, 40)_ = 0.44, *p* = 0.51, but a significant main effect of congruency, *F*_(1, 40)_ = 6.36, *p* < 0.02, $\eta_{p}^{2}$ = 0.15, and an alignment × congruency interaction, *F*_(1, 40)_ = 6.37, *p* < 0.02, $\eta_{p}^{2}$ = 0.15. When characters were aligned, the discrimination sensitivity in congruent trials was higher than that in incongruent trials (*p* < 0.001, Tukey-HSD corrected, Tukey, 1949), but when characters were misaligned there was no congruency effect (*p* > 0.4, Tukey-HSD corrected; Figure 3). As for reaction time, there was a significant main effect of congruency, *F*_(1, 40)_ = 18.51, *p* < 0.001, $\eta_{p}^{2}$ = 0.32, reaction time was shorter in the congruent trials than in the incongruent trials, but there was no main effect of alignment, *F*_(1, 40)_ = 2.25, *p* > 0.10, or an alignment × congruency interaction, *F*_(1, 40)_ = 2.71, *p* > 0.10, $\eta_{p}^{2}$ = 0.063 (Figure 4).

### Discussion

In studies on non-face categories with the CPc paradigm, a significant alignment × congruency interaction with a larger congruency effect in the aligned trials, is usually used as a mark of holistic processing (Bukach et al., 2006; Richler and Gauthier, 2014). We observed such an interaction under both durations in the “early-fixed” CPc paradigm, which suggested the involvement of holistic processing in visual word recognition. This is consistent with previous results in Leung (2012) and Wong et al. (2011, 2012), and in line with the findings which suggest the influence of “the whole” on recognizing “the part” in Chinese character recognition (Wang et al., 2003; Luo et al., 2010). Although the alignment × congruency interaction under two durations was obtained on different dependent variables—discrimination sensitivity under 170 ms and reaction time under 600 ms, we did not find a significant impact of stimuli exposure duration. Neither in discrimination sensitivity nor reaction time did we find a significant three-way interaction involving exposure duration, even when we used a very short duration of 170-ms. This meant the holistic word processing observed in the shorter duration was comparable to that in 600 ms. The results were in line with Richler et al. (2009a) where no change in holistic face processing was observed when the stimulus duration varied from 50 to 800 ms.

## The “Delayed-random cueing” task

In this section, we were to investigate if the abovementioned results could be replicated in the “delayed-random” task—another frequently-used version of the CPc paradigm. This informed us if the duration effect varied with experimental contexts.

### Participants and ethics

The same 82 participants in the “early-fixed” task participated in the current task. The participants who received the 170-ms “early-fixed” task were tested with the 170-ms “delayed-random” task, and those who received the 600-ms “early-fixed” task were tested with the 600-ms “delayed-random” task.

### Materials

To avoid the repetition effect of the stimuli, we used another set of 28 Chinese characters for the current task **(Appendix 2)**. Like in the “early-fixed” task, these characters were also of left-right configuration, and made from 14 common left radicals and 14 common right radicals with each radical used equally twice. They were comparable to the characters in the “early-fixed” task in terms of frequency, *t*_(54)_ = 0.12, *p* = 0.90 (Institute of Language Teaching and Research, 1986). Aligned and misaligned characters were made in the same way as in the “early-fixed” task.

### Procedure

The “delayed-random” task also consisted of four blocks, each block consisted of 56 trials. Each trial contained the following events sequentially, a fixation with random duration (1400 ms on average), a study character (with the cue), a mask (150 ms), a cue bracket (350 ms), and a test character (with the cue). The participants were required to make response within 1500 ms after the study character presentation. However, in this task the cue did not show up at the beginning of each block, it showed up only after the study character disappeared. Meanwhile, the cue position was randomized across trials, just as in the study by Wong et al. (2012). A practice session with at least 16 trials was also used prior to the formal test, and participants could not proceed to the formal test until they achieved at least 85% accuracy in practice. Characters used during practice were not used in the formal tests.

### Data analysis

The criteria for participant exclusion and trial exclusion were the same as in the “early-fixed” task. One participant was discarded because he did not finish the task, this left 81 participants for group analysis (31 males). For the remaining participants, 4.68% of the trials were discarded in the 600-ms task (41 participants), and 5.98% of the trials were discarded in the 170-ms task (40 participants).

### Results

#### Three-way ANOVA

**Figure 5:**
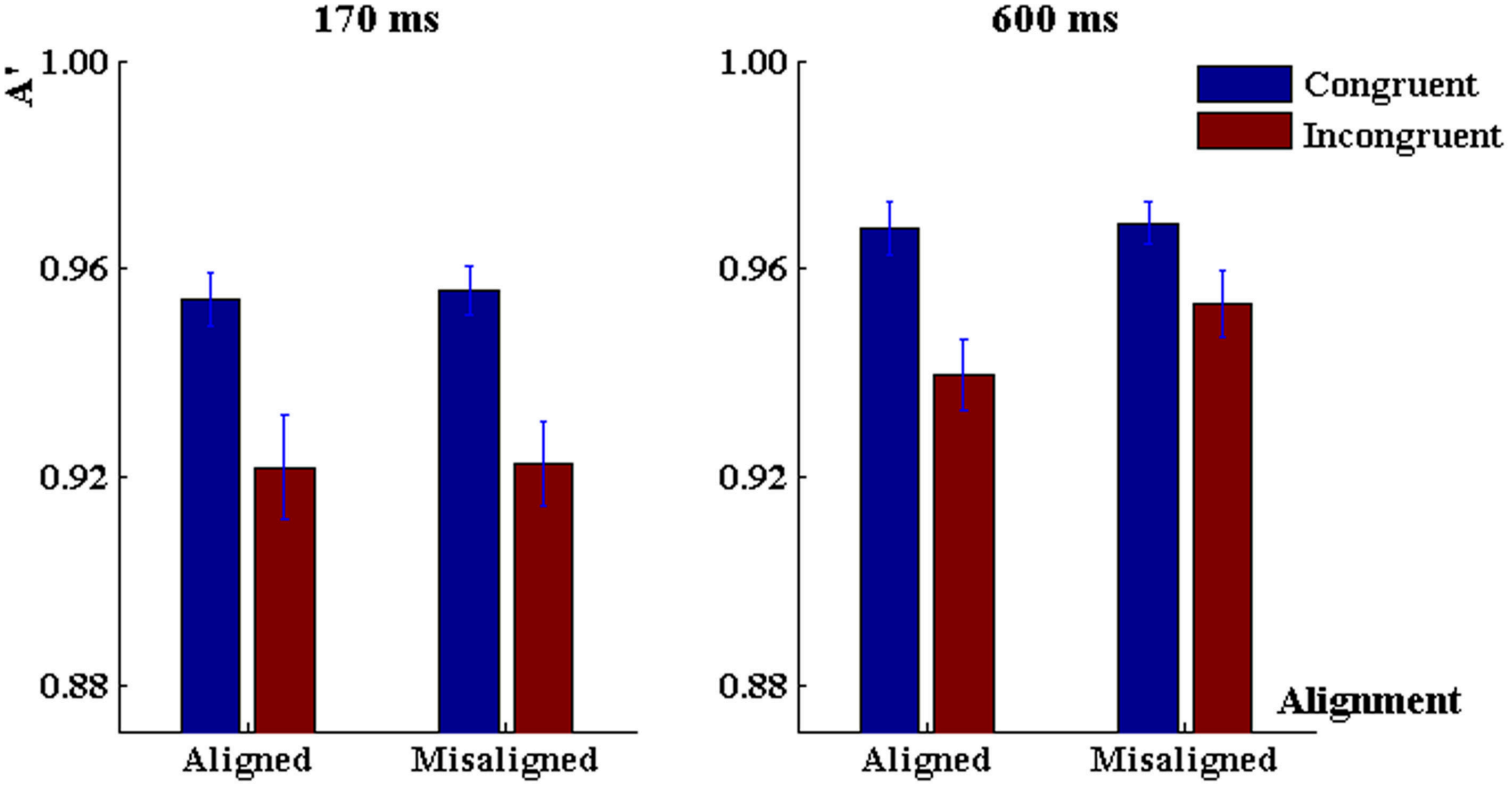
**Discrimination sensitivity in the “delayed-random cueing” task when character presentation was 170 ms (left panel) and 600 ms (right panel)**. Error bars represent 1 SEM.

The 2 × 2 × 2 ANOVA on discrimination sensitivity (Figure 5) identified significantly higher discrimination sensitivity in the 600-ms task than in the 170-ms task, *F*_(1, 79)_ = 9.07, *p* < 0.01, $\eta_{p}^{2}$ = 0.10, and higher sensitivity in the congruent than incongruent trials, *F*_(1, 79)_ = 23.15, *p* < 0.001, $\eta_{p}^{2}$ = 0.22. The exposure duration interacted with congruency, *F*_(1, 79)_ = 4.13, *p* < 0.05, $\eta_{p}^{2}$ = 0.05, but it did not interact with alignment, *F*_(1, 79)_ = 1.40, *p* > 0.20, $\eta_{p}^{2}$ = 0.02. Simple-effect analysis revealed the overall congruency effect was robust when characters were presented for 170 ms (*p* < 0.001, Tukey HSD), and marginally significant when the duration was 600 ms (*p* = 0.052, Tukey HSD). Importantly, there was no three-way interaction among duration, alignment and congruency, *F*_(1, 79)_ = 1.33, *p* > 0.20.

**Figure 6:**
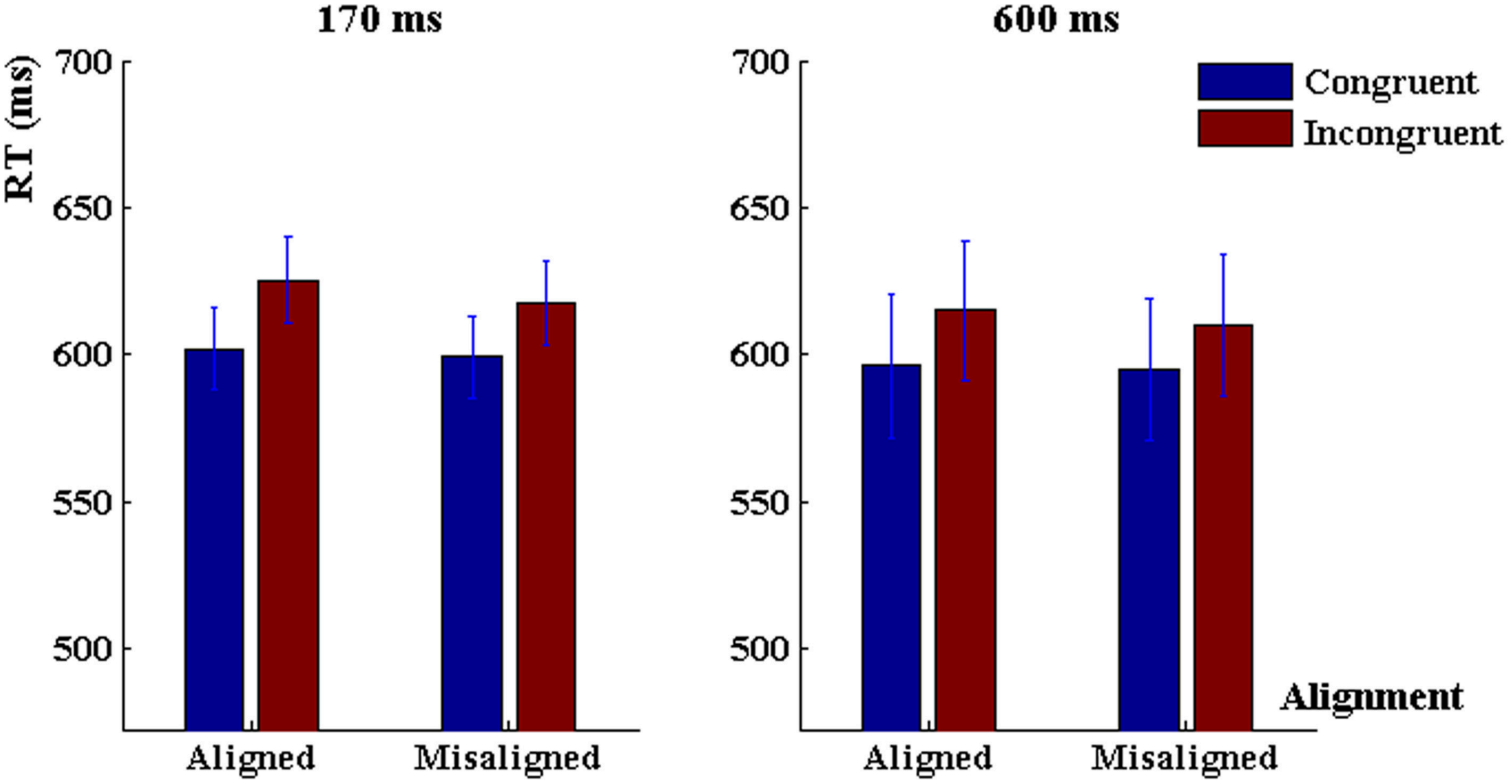
**Reaction time in the “delayed-random cueing” task when character presentation was 170 ms (left panel) and 600 ms (right panel)**. Error bars represent 1 SEM.

As for reaction time (Figure 6), there was a significant main effect of congruency, participants generally responded faster in the congruent trials, *F*_(1, 79)_ = 66.34, *p* < 0.001, $\eta_{p}^{2}$ = 0.46, there was a marginally significant main effect of alignment, *F*_(1, 79)_ = 3.87, *p* = 0.052, $\eta_{p}^{2}$ = 0.05. However, there was no main effect of exposure duration, *F*_(1, 79)_ = 0.06, *p* > 0.80, $\eta_{p}^{2}$ < 0.001. The exposure duration did not interact with alignment, *F*_(1, 79)_ = 0.23, *p* > 0.60, or congruency, *F*_(1, 79)_ = 0.77, *p* > 0.30, nor there was three-way interaction among duration, alignment and congruency, *F*_(1, 79)_ = 0.03, *p* > 0.80, $\eta_{p}^{2}$ < 0.001.

#### 600 ms

Analysis of the discrimination sensitivity revealed a significant main effect of congruency, *F*_(1, 40)_ = 31.57, *p* < 0.001, $\eta_{p}^{2}$ = 0.44, a main effect of alignment, *F*_(1, 40)_ = 4.45, *p* = 0.04, $\eta_{p}^{2}$ = 0.10, and a marginally significant interaction between alignment and congruency, *F*_(1, 40)_ = 3.32, *P* = 0.07, $\eta_{p}^{2}$ = 0.08. The overall discrimination sensitivity was higher in the congruent trials than in the incongruent trials. The congruency effect was significant both when the characters were aligned, *p* < 0.001, and when they were misaligned, *p* < 0.01, but much larger in the aligned trials. The alignment effect was significant when the trials were incongruent, *p* < 0.01 (Tukey-HSD), but not when they were congruent, *p* > 0.82 (Tukey-HSD). For reaction time, there was a robust congruency effect, *F*_(1, 40)_ = 35.06, *p* < 0.001, $\eta_{p}^{2}$ = 0.47, but no main effect of alignment, *F*_(1, 40)_ = 1.11, *p* > 0.30, nor alignment × congruency interaction, *F*_(1, 40)_ = 0.82, *p* > 0.30, $\eta_{p}^{2}\;=$ 0.02.

#### 170 ms

The overall discrimination sensitivity was higher in the congruent trials than in the incongruent trials, *F*_(1, 39)_ = 12.19, *p* = 0.001, $\eta_{p}^{2}$ = 0.23, but there was no main effect of alignment, *F*_(1, 39)_ = 0.15, *p* > 0.70, or alignment × congruency interaction, *F*_(1, 39)_ = 0.04, *p* > 0.80, $\eta_{p}^{2}$ = 0.001. Analysis on reaction time identified faster response in the congruent trials than the incongruent trials, *F*_(1, 39)_ = 32.33, *p* < 0.0001, $\eta_{p}^{2}$ = 0.45, but there was no main effect of alignment, *F*_(1, 39)_ = 2.99, *p* > 0.09, nor alignment by congruency interaction, *F*_(1, 39)_ = 0.59, *p* > 0.40, $\eta_{p}^{2}$ = 0.015.

### Discussion

When the stimuli were presented for 600 ms, there was a significant alignment by congruency interaction in discrimination sensitivity. This is consistent with the findings by Wong et al. (2012), especially their first experiment where the left-right characters were also used and presented sequentially. The alignment × congruency interaction was comparable between our “delayed-random” task and Wong et al.'s (2012), both in terms of the significance level (*p* = 0.07 in our study, and *p* = 0.067 in experiment 1 of Wong et al. (2012) and effect size ($\eta_{p}^{2}$: 0.08 vs. 0.104). This meant that, like in the “early-fixed” task, we could also observe holistic effect in the “delayed-random” task. Although we did not observe the alignment × congruency interaction when the characters were presented for 170 ms, statistically we did not observe a noticeable role of exposure duration on holistic word effect, as indicated by the non-significant three-way interaction when the exposure duration was involved. This means that though shortening the stimuli exposure would lead to larger general congruency effect, it does not necessarily change holistic word effect. The non-significant three-way interaction was consistent with the results in the “early-fixed” task, and provided converging evidence that the exposure duration brought about little change in the holistic word effect.

## Combined analysis of data in two tasks

Finally, we combined data in the “early-fixed” and “delayed-random” tasks to explore if cueing method interacted with exposure duration to modulate the holistic word effect. Four-way repeated measurements ANOVA of discrimination sensitivity revealed higher overall discrimination sensitivity in the 600-ms duration than in the 170-ms duration, *F*_(1, 79)_ = 6.15, *p* < 0.02, $\eta_{p}^{2}$ = 0.07, and higher discrimination sensitivity in the congruent trials than in the incongruent trials, *F*_(1, 79)_ = 28.68, *p* < 0.001, $\eta_{p}^{2}$ = 0.27. Moreover, there was a robust main effect of cueing method, *F*_(1, 79)_ = 75.17, *p* < 0.001, $\eta_{p}^{2}$ = 0.49, discrimination sensitivity was higher in the “early-fixed” cueing task than in the “delayed-random” task. The cueing method interacted significantly with duration, *F*_(1, 79)_ = 11.64, *p* < 0.002, $\eta_{p}^{2}$ = 0.13, and congruency, *F*_(1, 79)_ = 17.46, *p* < 0.001, $\eta_{p}^{2}$ = 0.18. The duration effect and the congruency effect were both larger in the “delayed-random” task than in the “early-fixed” task. Importantly, several findings about the alignment × congruency interaction were worth noting. First, the overall alignment × congruency interaction was significant, the congruency effect was larger in the aligned trials than in the misaligned trials, *F*_(1, 79)_ = 4.02, *p* < 0.05, $\eta_{p}^{2}$ = 0.05. Second, there was no cue × alignment × congruency interaction, *F*_(1, 79)_ = 0.43, *p* > 0.50, nor duration × alignment × congruency interaction, *F*_(1, 79)_ = 0.65, *p* > 0.40. Third, the four-way interaction among these factors was not significant, *F*_(1, 79)_ = 2.36, *p* > 0.10, $\eta_{p}^{2}$ = 0.029.

As for reaction time, four-way repeated measurements ANOVA revealed main effects of cueing method, *F*_(1, 79)_ = 92.41, *p* < 0.001, $\eta_{p}^{2}$ = 0.54, alignment, *F*_(1, 79)_ = 13.00, *p* < 0.001, $\eta_{p}^{2}$ = 0.14, and congruency, *F*_(1, 79)_ = 88.72, *p* < 0.001, $\eta_{p}^{2}$ = 0.53, but not duration, *F*_(1, 79)_ = 0.14, *p* > 0.70, $\eta_{p}^{2}$ = 0.002. Reaction time was shorter in the “early-fixed” cueing task, in aligned trials, and in congruent trials. Cueing method interacted significantly with congruency, *F*_(1, 79)_ = 20.85, *p* < 0.001, $\eta_{p}^{2}$ = 0.21, and marginally significantly with duration, *F*_(1, 79)_ = 20.85, *p* < 0.001, $\eta_{p}^{2}$ = 0.21. Meanwhile, the overall alignment × congruency interaction was significant, with larger congruency effect in the aligned trials than in the misaligned trials, *F*_(1, 79)_ = 7.87, *p* < 0.006, $\eta_{p}^{2}$ = 0.09. Second, there was no cue × alignment × congruency interaction, *F*_(1, 79)_ = 0.58, *p* > 0.40, nor duration × alignment × congruency interaction, *F*_(1, 79)_ = 0.08, *p* > 0.70. Third, the four-way interaction among these factors was not significant, *F*_(1, 79)_ = 0.25, *p* > 0.60, $\eta_{p}^{2}$ = 0.003. These accumulative evidence suggests that the holistic word effect was not modulated significantly by cueing method or duration.

## General discussion

In the current study, we investigated holistic word processing by the complete composite paradigm, which defined the holistic effect as the obligatory attention to all parts of the object despite participants being asked to focus only on one part (Gauthier and Bukach, 2007; Wong et al., 2012). We explored if and how the holistic word effect would vary as a function of stimuli exposure duration, under two versions of the complete composite paradigm (the CPc paradigm): the “early-fixed” task where the cue showed up early in each trial at a fixed location, and the “delayed-random” task where the cue showed up only after the study character at randomized locations. We observed holistic word processing in both tasks. This is inconsistent with the argument that visual words are processed in a piecemeal manner (Farah, 1990; Wilson and Taylor, 2009), but in line with the claim that visual words are processed holistically (Allen et al., 1995; Luo et al., 2010; Wong et al., 2012), and consistent with previous findings that holistic processing, at least when it was measured by the CPc paradigm, could be observed in non-face categories (Wong and Gauthier, 2010; Richler and Gauthier, 2013).

The current study, which explored the association between the holistic word effect and stimuli exposure duration, helped characterize the dynamics of holistic word processing. An important finding in this study was that variation in the exposure duration did not bring about significant change in holistic word effect, at least when the stimuli was presented in the range of around 200 to 600 ms. The holistic word effect under the 170-ms duration was not different from that under the 600-ms duration. The non-significance of exposure duration was observed in the “early-fixed” task where the attentional locus was notified early and clearly, and in the “delayed-random” task where the task was more challenging. This means that expert readers could grasp global information within a fairly short interval. Meanwhile, it echoes the findings of Richler et al. (2009b) where no duration effect was observed on holistic face processing. The absence of duration effect in these two categories suggests that the holistic processing in visual words and faces may share some common principles (Cao et al., 2014a,b).

Some word recognition models assume that words are initially formed from component letters (McClelland, 1976; Adams, 1979). According to these models, it may take time for holistic word representation to manifest. But recent progress suggests that the “whole representation” of visual words could be achieved very early. For example, studies using the electroencephalogram technique find that the N170 (“recognition potential” in some articles, Zhang et al., 2009), a component of the event-related potential (ERP) which shows increased negativity 130–200 ms after stimulus onset, repesents a logographic processing strategy in visual word recognition (Simon et al., 2007; Cao and Zhang, 2011). In Chinese linguistic system, the N170 shows face-like inversion effect (Wang et al., 2011), and fast adaptation (Cao et al., 2014b) for printed Chinese characters. A recent ERP study suggests that holistic word representation may occur much earlier. Chen et al. (2013) asked participants to compare the top parts of two sequential Chinese characters in the CPc task. They found that when the two top parts were identical, the P1-an ERP component starting about 80 ms after word presentation-showed a larger amplitude when the top targets were accompanied by two different bottom halves. This effect was observed only when the top and bottom halves were aligned but not when misaligned. These collective results suggest that visual words could be represented holistically at a perceptual level.

The results in our study also have methodological implications. Holistic processing is a central concept for understanding object recognition (Richler et al., 2012; Rossion, 2013; Richler and Gauthier, 2014; Watson and Robbins, 2014), and the two versions of CPc paradigm has been frequently used as its measurements it in the literature. Our study observed lower discrimination sensitivity and larger overall congruency effect in the “delayed-random” task than in the “early-fixed” task. Crucially, we observed reliable alignment by congruency interaction in the “early-fixed” tasks, but in the “delayed-random” tasks the interaction was not as reliable. Since the alignment × congruency interaction is a better marker of holistic processing in non-face categories (Richler et al., 2009a; Wong et al., 2009b; Richler and Gauthier, 2014), our results suggest that at least in visual word recognition, the “early-fixed” version of the CPc paradigm is not inferior to the “delayed-random” version in terms of the sensitivity to observe holistic effect. Given the “early-fixed” task involves less attentional switching and its procedure is easy to explain, it could be a better choice when we are to carry out the CPc tasks on special participants like children, old people or those who are illiterate.

These results are also informative for designing experiments with the composite task. In psychological studies, researchers usually shorten the exposure duration to alleviate the ceiling effect (Rothschild et al., 1990; Tanaka et al., 2012). In our study, even when the duration was as brief as 170 ms, the participants (expert Chinese readers) still achieved a very high level of discrimination sensitivity. The average discrimination index was 0.984 in the 170 ms-“early-fixed” task, and 0.929 in the 170 ms-”delayed-random” task. Meanwhile, we did not observe a significant decrease in the holistic word effect when the duration was shortened. This suggests that when investigating holistic word effect using the CPc paradigm, probably it is not an optimal choice to merely shorten the stimuli exposure duration when a ceiling effect is imminent. This was not at odds with those in Wong et al. (2012) and Hsiao and Cottrell (2009), but instead reflected the necessity to combine other strategies, such as to use a challenging response deadline and stimuli contrast level as they did in their studies. In Wong's study (Wong et al., 2012) when expert Chinese readers were required to respond within 700 ms, the average discrimination sensitivity (*A*') decreased to around 0.90.

Finally, it is worth noting that the current study used only two levels of exposure duration, it did not use a much shorter presentation such as 17 or 50 ms in Richler's study, nor a much longer duration such as 1200 ms in Hole (1994). Therefore, it is unknown if our results characterize holistic word processing under these extreme durations. Meanwhile, during the past decades a variety of paradigms have been developed to explore holistic processing and each paradigm holds its own interpretation about the term “holistic processing,” currently there is still a lack of consensus on the theoretical constructs of “holistic processing” (Piepers and Robbins, 2012; Richler et al., 2012; Watson and Robbins, 2014). In the current study we investigated holistic word processing using the CPc paradigm. We respect the merits of other representative paradigms such as the part-whole task (Tanaka and Farah, 1993) and the inversion task (Yin, 1969) in exploring the whole-part relationship in face and object recognition. The readers should be aware that findings here perhaps could not be generalized to other paradigms. Further studies could be conducted to explore the holistic word effect with those paradigms, which can provide multifaceted information and contribute to universal models of object processing.

## Author contributions

CC, HL, and JC designed this study. CC, NA, and SS collected data. CC, NA, HL, and JC analyzed data and wrote manuscript. CC, NA, SS, HL, and JC made revisions and approved the submission.

### Conflict of interest statement

The authors declare that the research was conducted in the absence of any commercial or financial relationships that could be construed as a potential conflict of interest.

## Appendix 1: characters used in the “early-fixed” task

驰驴沪没般航吭呕枢杯坏址扯抬怡悦锐钵体他绽纱妙娱误诣脂腚

## Appendix 2: characters used in the “delayed-random” task

徒彼跛跃袄衬时晒牺牧敌刮则贴粘粗狙狠限陡炽烛蚀饮软轶秩积
